# Supplementary material for: Broad phosphorylation mediated by testis-specific serine/threonine kinases contributes to spermiogenesis and male fertility
Source: Nat Commun. 2023 May 6;14:2629. doi: 10.1038/s41467-023-38357-0 (PMC10164148; doi:10.1038/s41467-023-38357-0)
Supplement: Supplementary file 1 — Supplementary information [file 41467_2023_38357_MOESM1_ESM.pdf]

Supplementary information

Broad phosphorylation mediated by testis-specific serine/threonine kinases contributes to spermiogenesis and male fertility

Xuedi Zhang[1#], Ju Peng[1#], Menghua Wu[1,2#], Angyang Sun[1], Xiangyu Wu[1], Jie Zheng[1], Wangfei Shi[1], Guanjun Gao[1*]

Author affiliations

[1] School of Life Science and Technology, ShanghaiTech University, Shanghai 201210, China
[2] School of Life Sciences, Tsinghua University, Beijing, 100084, China

This PDF file includes:

Supplementary Fig. S1 to S16

Supplementary Table S1

Other Supplementary Files for this manuscript include the following:

Supplementary Data 1 to 4

a

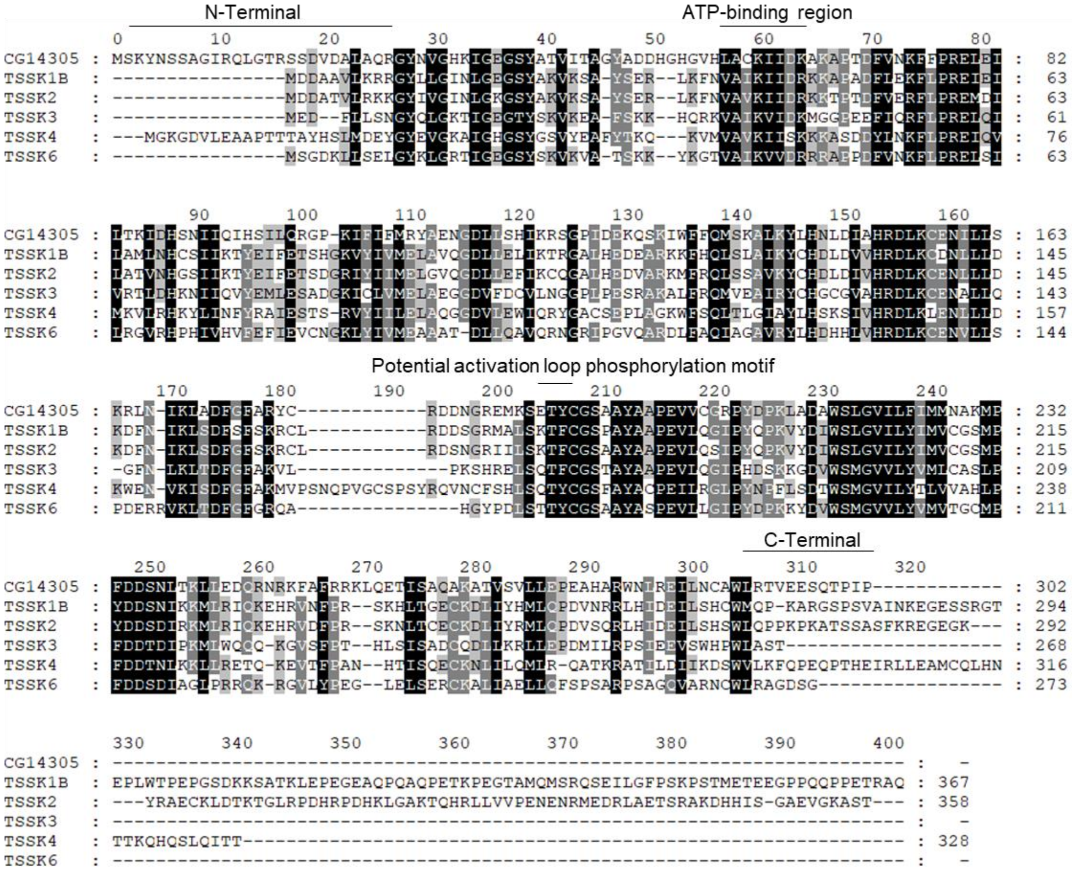

b

	Identity	Similarity
TSSK1B	46%	66%
TSSK2	46%	66%
TSSK3	42%	62%
TSSK4	37%	54%
TSSK6	42%	60%

c

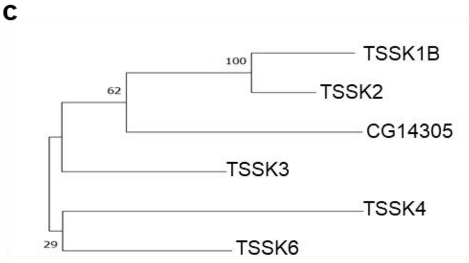

Supplementary Fig. S1. *Drosophila* CG14305 encodes an ortholog of human TSSKs

a Multiple sequence alignment of the protein sequence of *Drosophila* CG14305 with those of five human TSSKs (TSSK1B, TSSK2, TSSK3, TSSK4, and TSSK6) using ClustalW. Conserved regions such as the ATP-binding region and potential activation loop phosphorylation motif are indicated. **b** Protein identities and similarities between *Drosophila* CG14305 and five human TSSKs analyzed by Blastp. **c** Phylogenetic analysis of *Drosophila* CG14305 and five human TSSKs using Phylogeny.

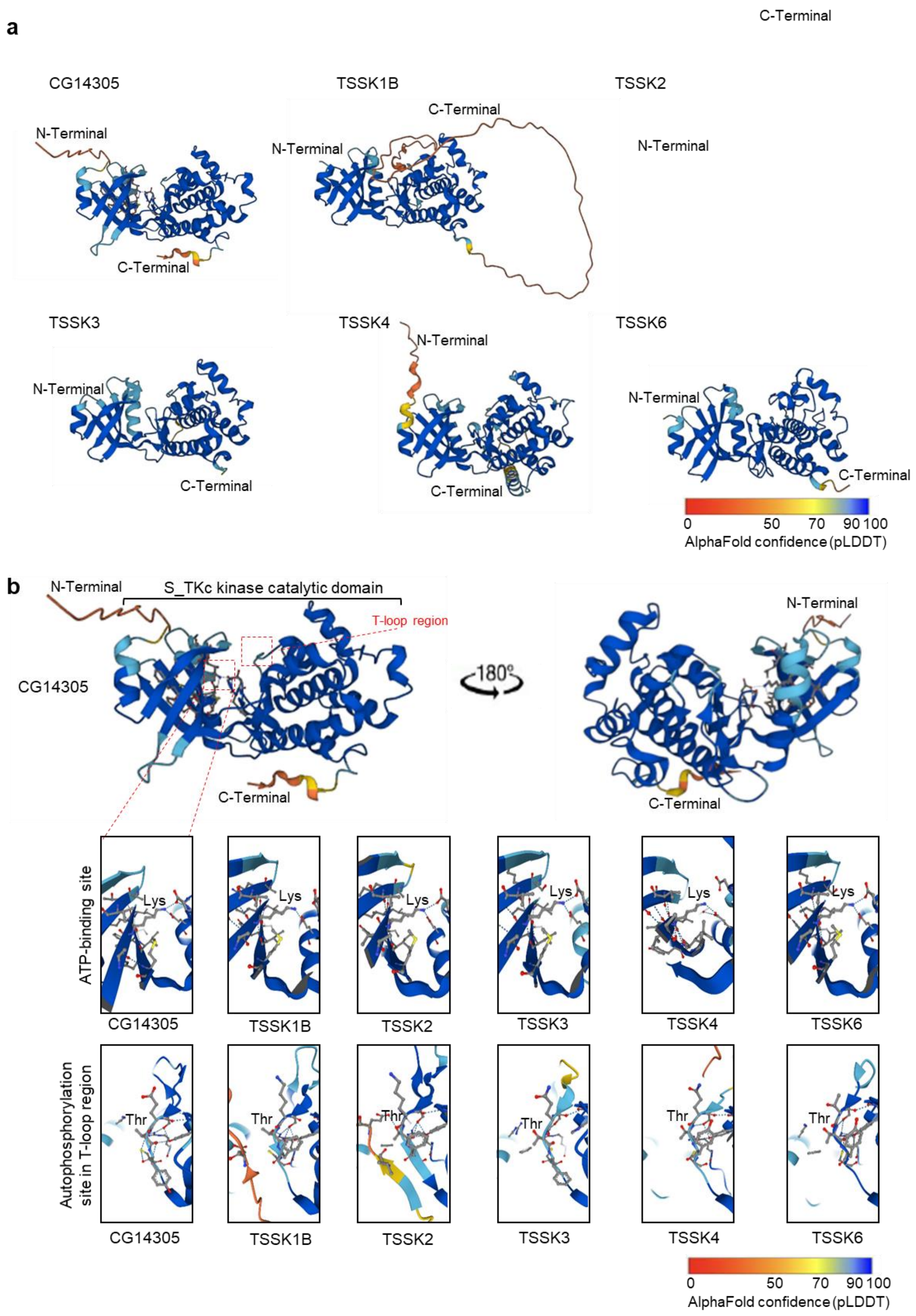

Supplementary Figure S2. *Drosophila* CG14305 encodes an ortholog of human TSSKs

a Protein structures of *Drosophila* CG14305 and five human TSSKs predicted in the AlphaFold Protein Structure Database showing conservation of the S_TKc kinase catalytic domain and disordered N- and C-terminal domains. The AlphaFold model is colored according to the AlphaFold confidence score (pLDDT). **b** Enlargement of the ATP-binding site and autophosphorylation site in the T-loop region. AlphaFold Structure Prediction showing the conserved lysine residue in the ATP-binding site and the conserved threonine residue in the T-loop region in CG14305 and five human TSSKs.

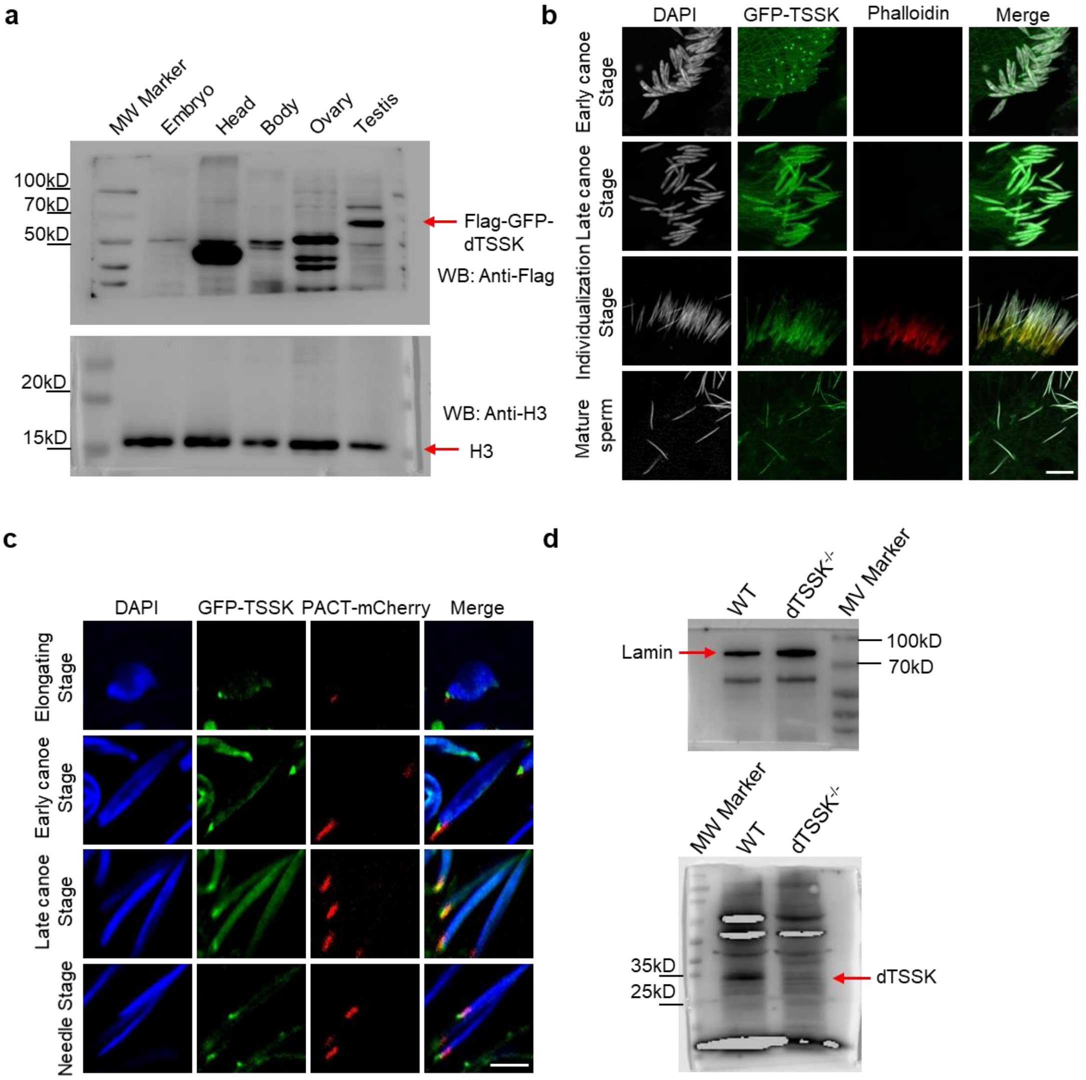

Supplementary Figure S3. Expression and localization of dTSSK in *Drosophila*

a Raw image of western blot in Figure 1a. **b** IF showing the localization of dTSSK at different stages of spermiogenesis in *Drosophila*. DNA stained with DAPI, white; GFP-dTSSK, green; individualization complex (IC), red. Scale bar, 10 μm. **c** Live imaging showing the colocalization (white arrowheads) of dTSSK and PACT (targeting the basal body) at different stages of spermiogenesis in *Drosophila*. DNA stained with DAPI, blue; GFP-dTSSK, green; PACT-mCherry, red. Scale bar, 5 μm. **d** Raw images of western blot in Figure 1e.

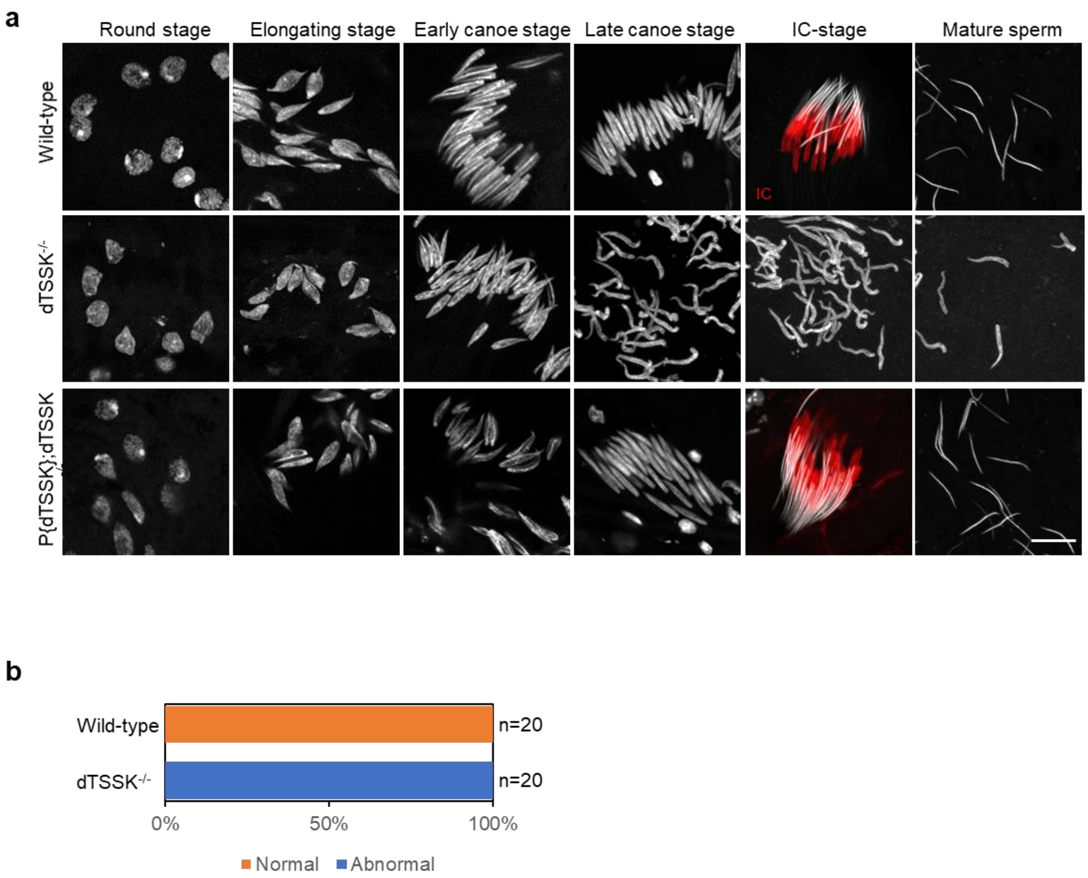

Supplementary Fig. S4. dTSSK depletion causes severe spermiogenesis defects
a Spermatid nuclear morphology at different stages of spermiogenesis in wild-type (w^{1118}), dTSSK$^{-/-}$, and dTSSK-rescued dTSSK$^{-/-}$ flies. IC staining shows that dTSSK depletion results in failure of sperm individualization. DNA stained with DAPI, white; IC stained with phalloidin, red. Scale bar, 10 μm. **b** Morphological statistics of sperm flagella in wild-type and dTSSK mutant *Drosophila*. 20 sperm bundles (64-cells spermatid) were analyzed.

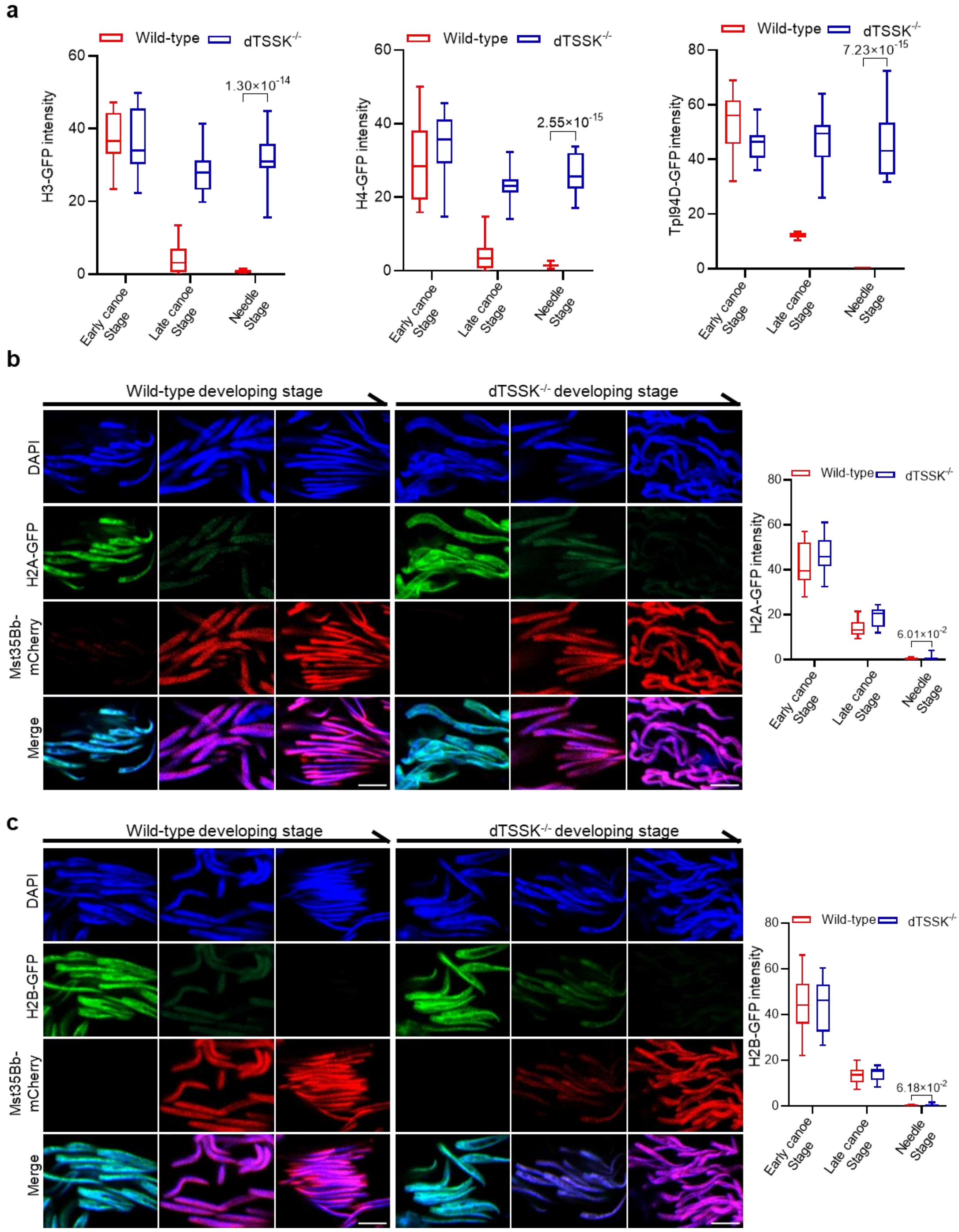

Supplementary Fig. S5. dTSSK depletion impairs histone to protamine transition

a Quantitative analysis of fluorescence intensity for H3-GFP, H4-GFP and Tpl94D-GFP in sperm nuclei. 15 sperm cells (n = 15) were analyzed at each stage (paired t-test). **b** and **c** Live imaging showing the normal removal of histones H2A (**b**) and H2B (**c**) from sperm DNA in dTSSK$^{-/-}$ flies. w^{1118} flies were used as the wild type. Histones H2A and H2B were labeled with GFP, and the protamine Mst35Bb was labeled with mCherry. Sperm images were mainly collected according to the developmental stages of spermatozoa. DNA stained with DAPI, blue; H2A-GFP and H2B-GFP, green; Mst35Bb-mCherry, red. Scale bar, 5 μm. 15 sperm cells (n = 15) were quantitatively analyzed at each stage. Statistical analyses were performed by two-sided Student's t-test and p values are indicated on the box plots. Center line: median. Box limits: upper and lower quartiles. Whiskers: minima and maxima. Source data are provided as Source Data file.

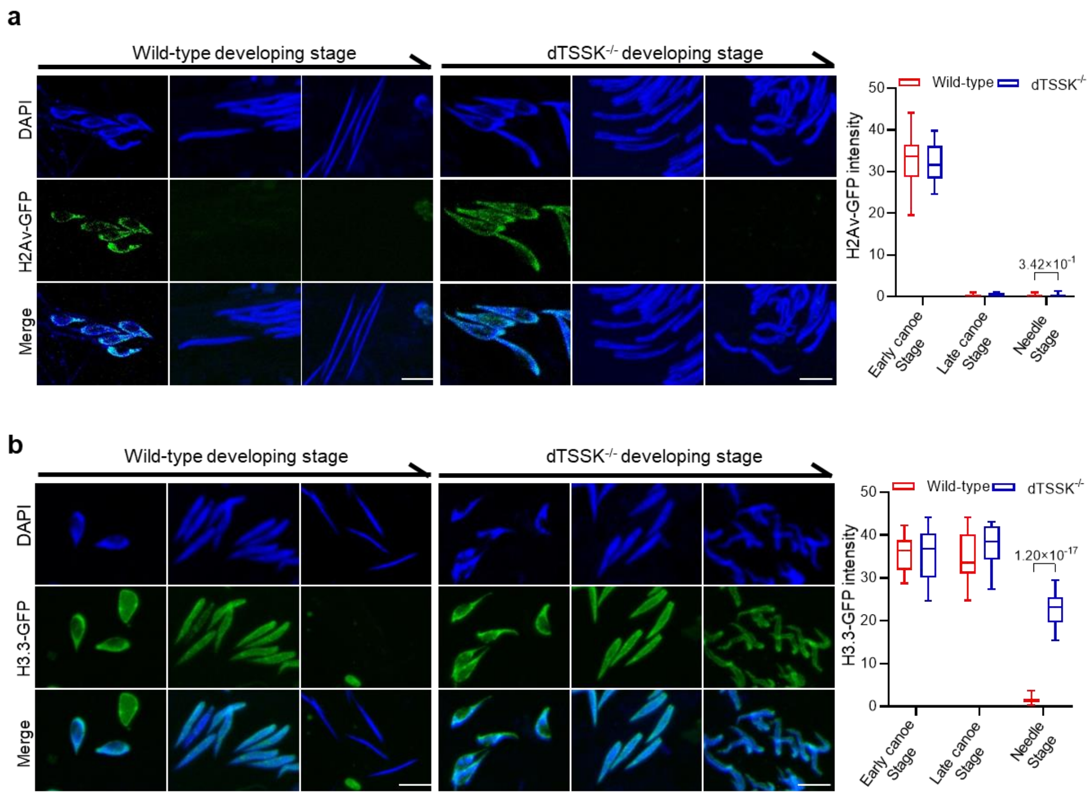

Supplementary Fig. S6. dTSSK depletion impairs removal of H3.3 from sperm DNA

a Live imaging showing the normal removal of the histone variant H2Av from sperm DNA in dTSSK$^{-/-}$ flies. w^{1118} flies were used as the wild type. H2Av was labeled with GFP. DNA stained with DAPI, blue. Scale bar, 5 μm. 15 sperm cells (n = 15) were quantitatively analyzed at each stage (paired t-test). **b** Live imaging showing defective removal of the histone H3 variant H3.3 from sperm DNA in dTSSK$^{-/-}$ flies. w^{1118} flies were used as the wild type. H3.3 was labeled with GFP. DNA stained with DAPI, blue. Scale bar, 5 μm. 15 sperm cells (n = 15) were quantitatively analyzed at each stage. Statistical analyses were performed by two-sided Student's t-test and p values are indicated on the box plots. Center line: median. Box limits: upper and lower quartiles. Whiskers: minima and maxima. Source data are provided as Source Data file.

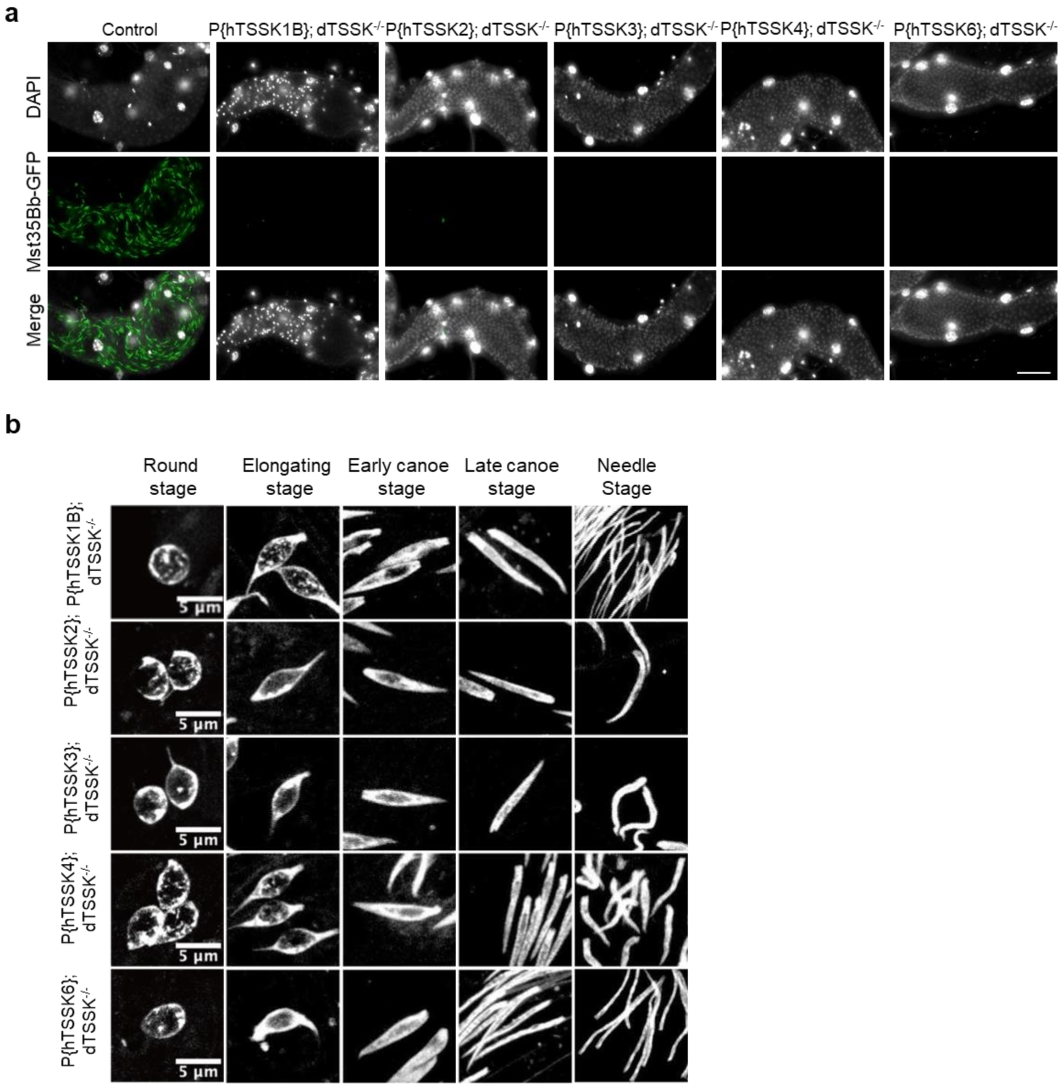

Supplementary Fig. S7. Sperm nuclear morphology in human TSSK-rescued dTSSK$^{-/-}$ flies

a Live imaging of seminal vesicles in the indicated genotype stained with DAPI (white). Sperm nuclei (green) were labeled with Mst35Bb-GFP. Scale bar, 50 μm. **b** Sperm nuclear morphology at different stages in human TSSK-rescued dTSSK$^{-/-}$ flies. DNA stained with DAPI, white. Scale bar, 5 μm.

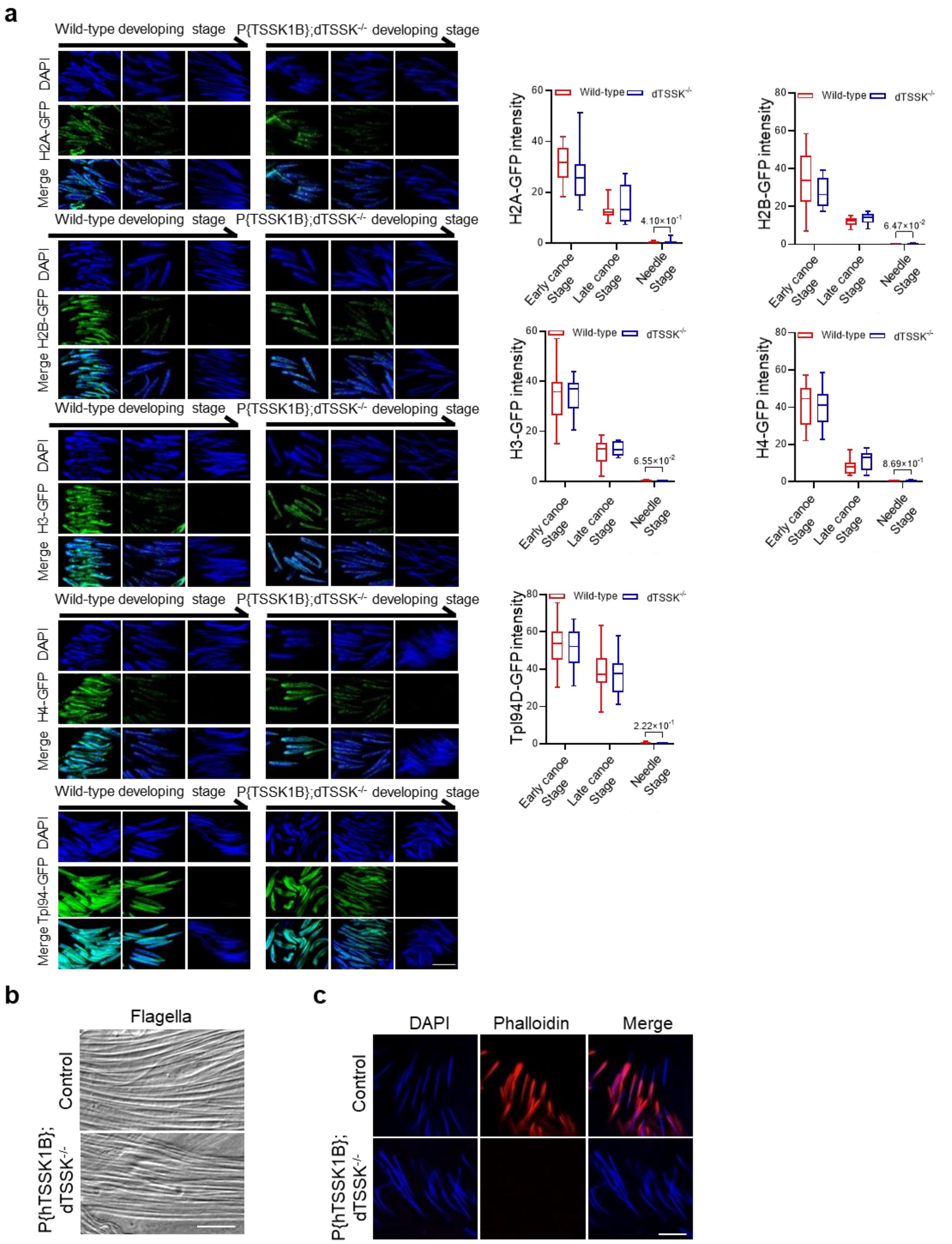

Supplementary Fig. S8. Sperm nuclear morphology analysis of human TSSK-rescued dTSSK$^{-/-}$ flies

a Live imaging showing the removal of histones H2A, H2B, H3, and H4 and the transition protein Tpl94D from sperm DNA in human TSSK1B-rescued dTSSK$^{-/-}$ flies. Wild-type flies (w^{1118}) were used as the control. H2A, H2B, H3, H4, and Tpl94D were labeled with GFP. Sperm images were mainly collected according to the progressive stages of sperm development. DNA stained with DAPI, blue; H2A-GFP, H2B-GFP, H3-GFP, H4-GFP, and Tpl94D-GFP, green. Scale bar, 5 μm. 15 sperm cells (n = 15) were quantitatively analyzed at each stage. Statistical analyses were performed by two-sided Student's t-test and p values are indicated on the box plots. Center line: median. Box limits: upper and lower quartiles. Whiskers: minima and maxima. **b** Flagellar morphology in wild-type (w^{1118}), dTSSK$^{-/-}$, and human TSSK1B-rescued dTSSK$^{-/-}$ flies.

Scale bar, 10 μm. **c** IF showing that human TSSK1B rescues the failure of IC formation in $dTSSK^{-/-}$ flies. w^{1118} flies were used as a control. ICs stained with phalloidin, red. Nuclei stained with DAPI, blue. Scale bar, 10 μm. Source data are provided as Source Data file.

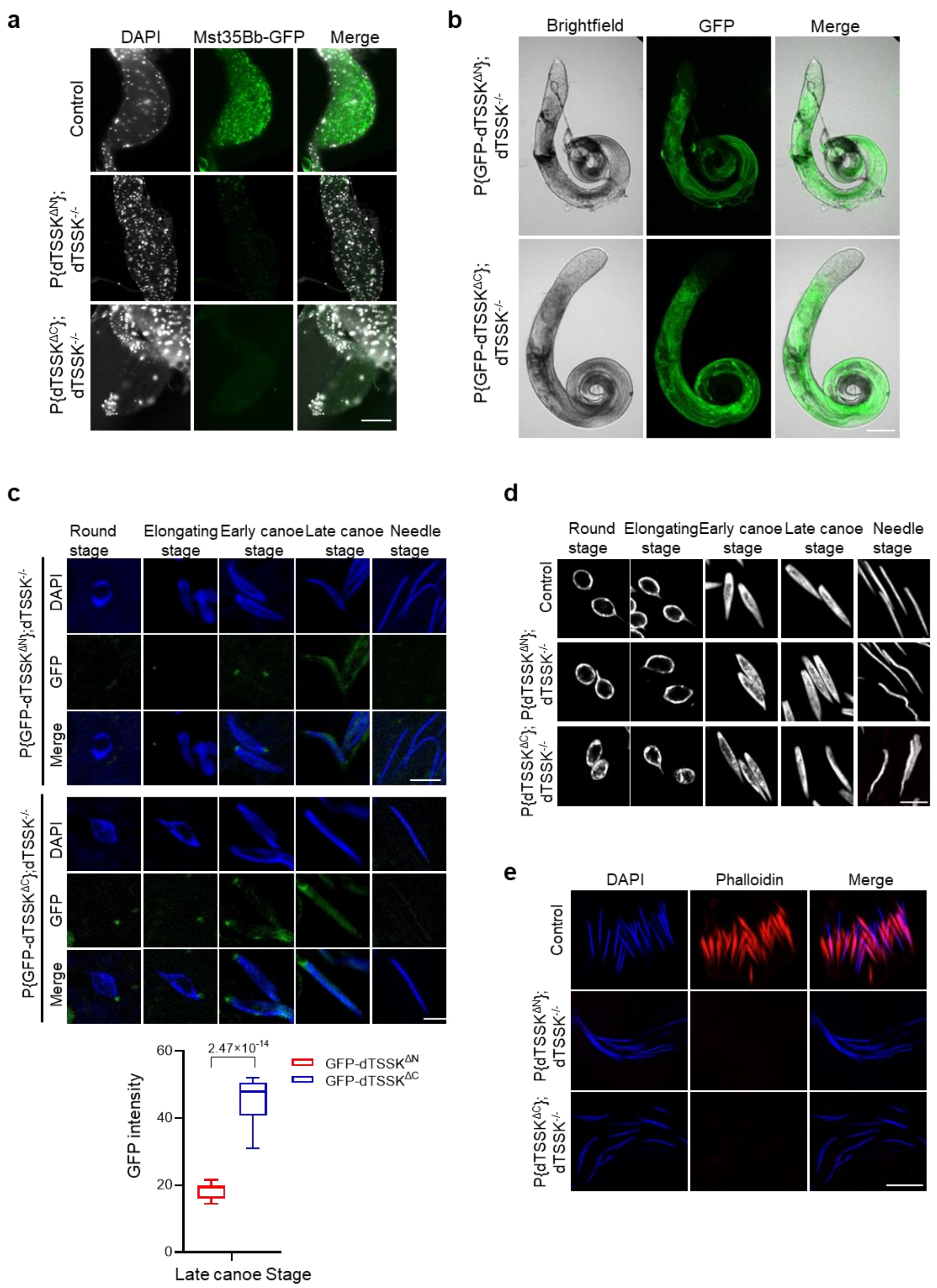

Supplementary Fig. S9. The N- and C-terminal domains of dTSSK are both essential for male fertility

a Images of seminal vesicles in the indicated genotype stained with DAPI (white). Sperm nuclei (green) were labeled with Mst35Bb-GFP. No sperm are detected in $dTSSK^{\Delta N}$ and $dTSSK^{\Delta C}$ flies. Scale bar, 50 μm. **b** Live imaging showing the distributions of $dTSSK^{\Delta N}$ (GFP-$dTSSK^{\Delta N}$) and $dTSSK^{\Delta C}$ (GFP-$dTSSK^{\Delta C}$) proteins in whole testes. DNA stained with DAPI, white; GFP-$dTSSK^{\Delta N}$ and GFP-$dTSSK^{\Delta C}$, green. Scale bar, 100 μm. **c** Live imaging showing the distributions of $dTSSK^{\Delta N}$ (GFP-$dTSSK^{\Delta N}$) and $dTSSK^{\Delta C}$ (GFP-$dTSSK^{\Delta C}$) proteins in spermiogenesis. DNA stained with DAPI, blue; GFP-$dTSSK^{\Delta N}$ and GFP-$dTSSK^{\Delta C}$, green. Scale bar, 5 μm. 15 sperm cells (n = 15) were quantitatively analyzed at each stage. Statistical analyses were performed by two-sided Student's t-test and p values are indicated on the box plots. Center line: median. Box limits: upper and lower quartiles. Whiskers: minima and maxima.

d Sperm nuclear morphology at different stages of spermiogenesis in wild-type (w^{1118}), $dTSSK^{\Delta N}$, and $dTSSK^{\Delta C}$ flies. DNA stained with DAPI, white. Scale bar, 5 µm. **e** IF showing the failure of IC formation in $dTSSK^{\Delta N}$ and $dTSSK^{\Delta C}$ flies. w^{1118} flies were used as a control. ICs stained with phalloidin, red. Sperm nuclei stained with DAPI, blue. Scale bar, 10 µm. Source data are provided as Source Data file.

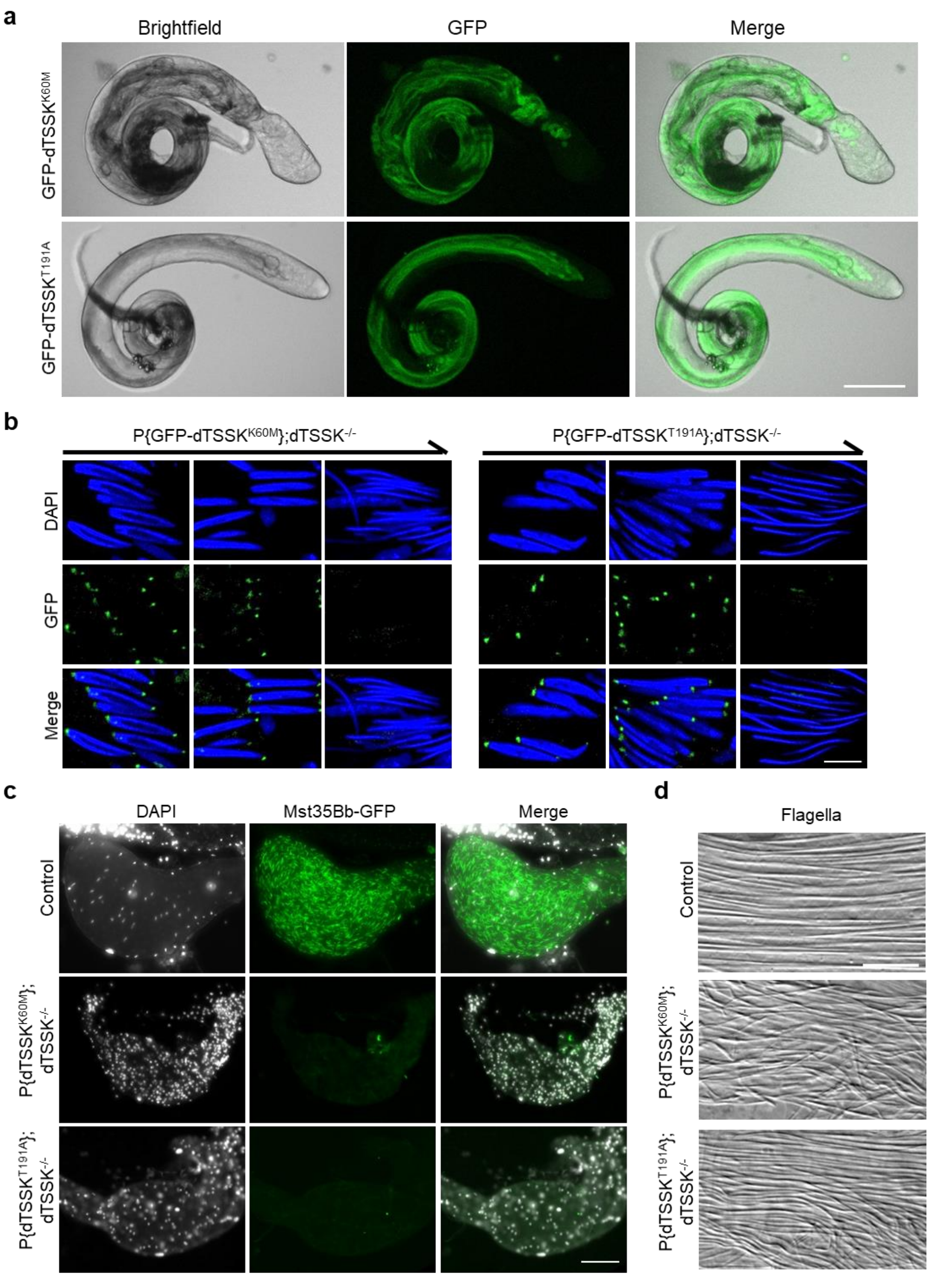

Supplementary Fig. S10. Kinase activity of dTSSK is essential for spermiogenesis
a Distributions of dTSSK^{K60M} (GFP-dTSSK^{K60M}) and dTSSK^{T191A} (GFP-dTSSK^{T191A}) proteins in testes. GFP-dTSSK^{K60M} and GFP-dTSSK^{T191A}, green. Scale bar, 100 μm. **b** Distributions of dTSSK^{K60M} (GFP-dTSSK^{K60M}) and dTSSK^{T191A} (GFP-dTSSK^{T191A}) proteins in sperm at different stages of development. Mutations of Lys60 or Thr191 lead to failure of localization in sperm DNA. DNA stained with DAPI, blue; GFP-dTSSK^{K60M} and GFP-dTSSK^{T191A}, green. Scale bar, 5 μm. **c** Images of seminal vesicles in the indicated genotype stained with DAPI (white). Sperm nuclei (green) were labeled with Mst35Bb-GFP. No sperm are detected in dTSSK^{K60M} and dTSSK^{T191A} flies. Scale bar, 50 μm. **d** Flagellar morphology in wild-type, dTSSK^{K60M}, and dTSSK^{T191A} flies. Scale bar, 10 μm. Lys60 or Thr191 mutation in dTSSK slightly affects sperm flagella alignment. Scale bar, 10 μm.

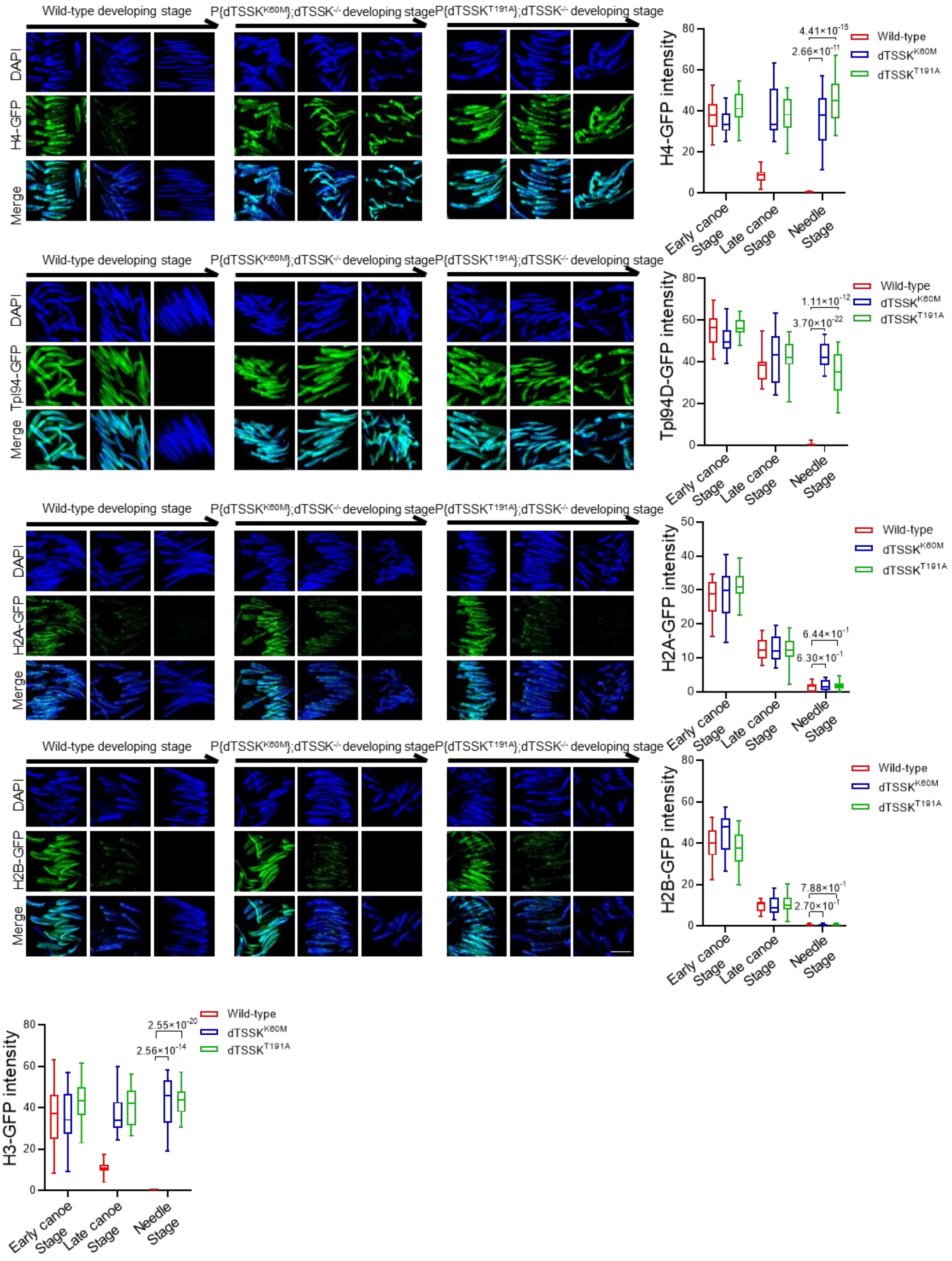

Supplementary Fig. S11. Kinase activity of dTSSK is essential for histone to protamine transition

Distributions of histone (H2A-GFP, H2B-GFP, and H4-GFP) and transition protein-like 94D (Tpl94D-GFP) proteins in sperm nuclei of wild-type (w^{1118}), dTSSK^{K60M}, and dTSSK^{T191A} flies. DNA stained with DAPI, blue; H2A-GFP, H2B-GFP, H4-GFP, and Tpl94D-GFP, green. Scale bar, 5 μm. Quantitative analysis of fluorescence intensity for H4-GFP, Tpl94D-GFP, H2A-GFP, H2B-GFP, and H3-GFP in sperm nuclei of wild-type (w^{1118}), dTSSK^{K60M}, and dTSSK^{T191A} flies. 15 sperm cells ($n = 15$) were analyzed at each stage. Statistical analyses were performed by two-sided Student's t-test and p values are indicated on the box plots. Center line: median. Box limits: upper and lower quartiles. Whiskers: minima and maxima. Source data are provided as Source Data file.

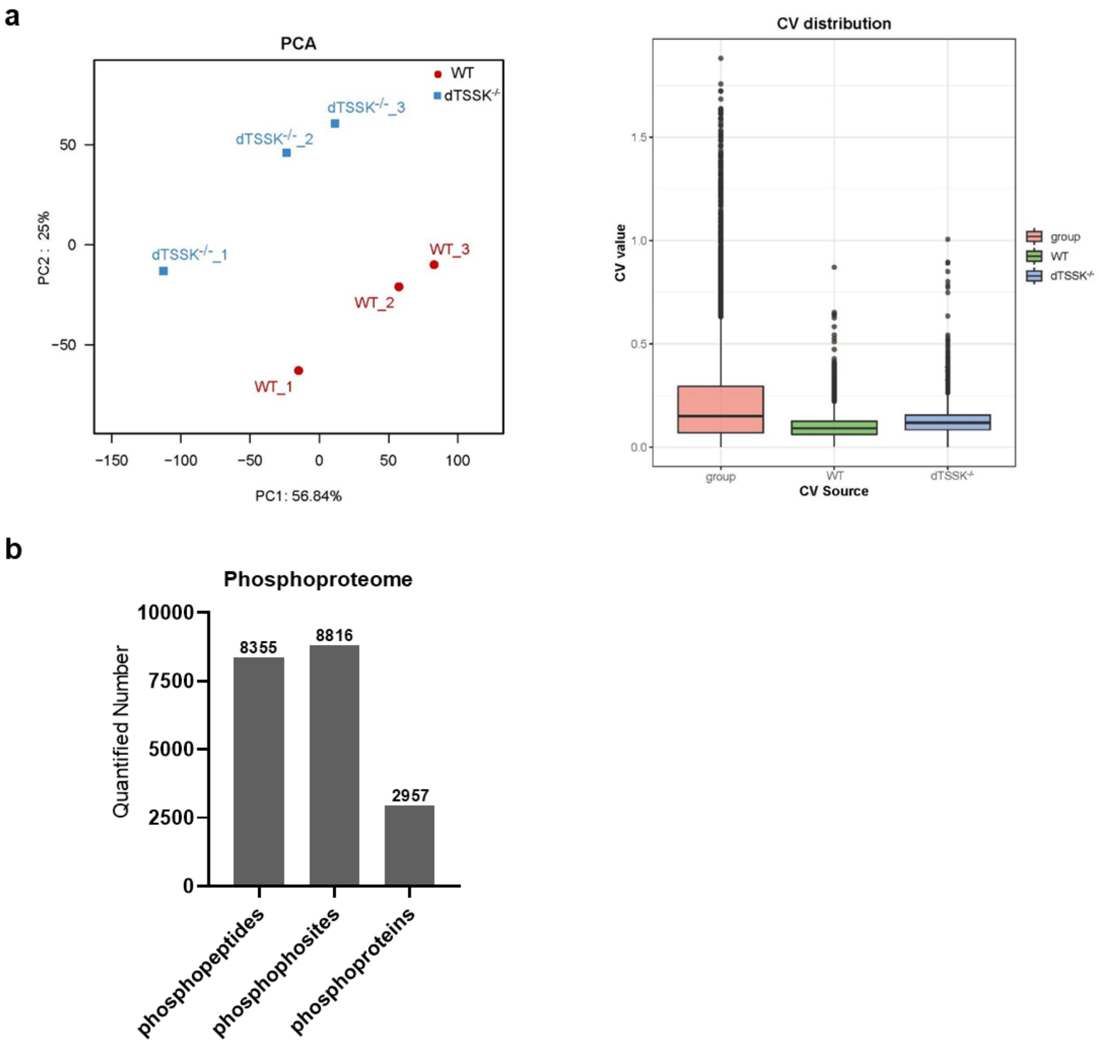

Supplementary Fig. S12. Phosphoproteomic screening identifies physiological substrates of dTSSK

a Principal component analysis of the TMT-based phosphoproteome and a boxplot of the coefficient of variation (CV) of protein distribution in wild-type and dTSSK$^{-/-}$ flies (n = 3 per group). Center line: median. Box limits: upper and lower quartiles. Whiskers: minima and maxima. **b** Quantified number statistics in the phosphoproteome.

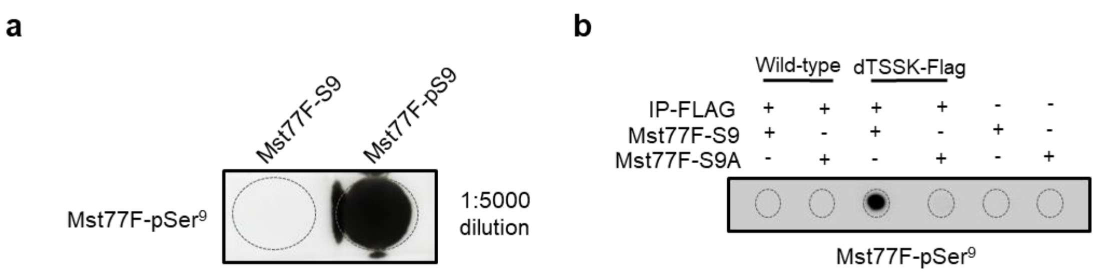

Supplementary Fig. S13. Identification of Mst77F as a substrate of dTSSK in vitro and in vivo

a Dot blot showing that the anti-Mst77F-pSer^9 antibody specifically recognizes phosphorylated Mst77F-Ser9. **b** Assessment of dTSSK-mediated Mst77F-Ser9 phosphorylation by an in vitro kinase assay followed by dot blotting. Peptides corresponding to Mst77F-Ser9 are phosphorylated by purified dTSSK-Flag and detected with an anti-Mst77F-pSer^9 antibody. Source data are provided as Source Data file.

a

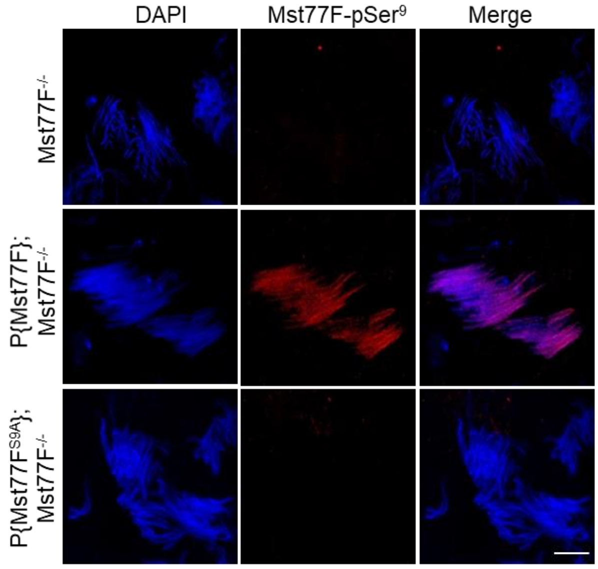

b

Genotype	Fertility ♂	Fertility ♀
w;+/+	+	+
w; Mst77F$^{-/-}$	-	+
W;P{Mst77F};Mst77F$^{-/-}$	+	+
W;P{Mst77F^{S9A}};Mst77F$^{-/-}$	+	+

c

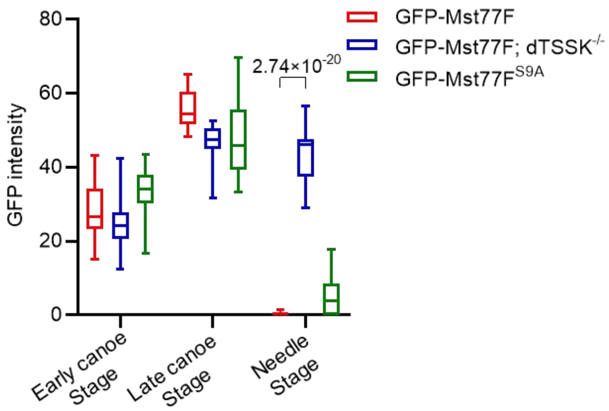

Supplementary Fig. S14. Phosphorylation of Mst77F by dTSSK contributes to spermiogenesis

a IF showing phosphorylated Mst77F-Ser9 (red) in Mst77F$^{-/-}$ and Mst77F- and Mst77F^{S9A}-rescued Mst77F$^{-/-}$ flies. Sperm nuclei stained with DAPI, blue. Scale bar, 10 μm. **b** Fertility testing of wild-type and Mst77F$^{-/-}$ male and female flies. **c** Quantitative analysis of fluorescence intensity for GFP-Mst77F in sperm nuclei. 15 sperm cells (n = 15) were analyzed at each stage. Statistical analyses were performed by two-sided Student's t-test and p values are indicated on the box plots. Center line: median. Box limits: upper and lower quartiles. Whiskers: minima and maxima. Source data are provided as Source Data file.

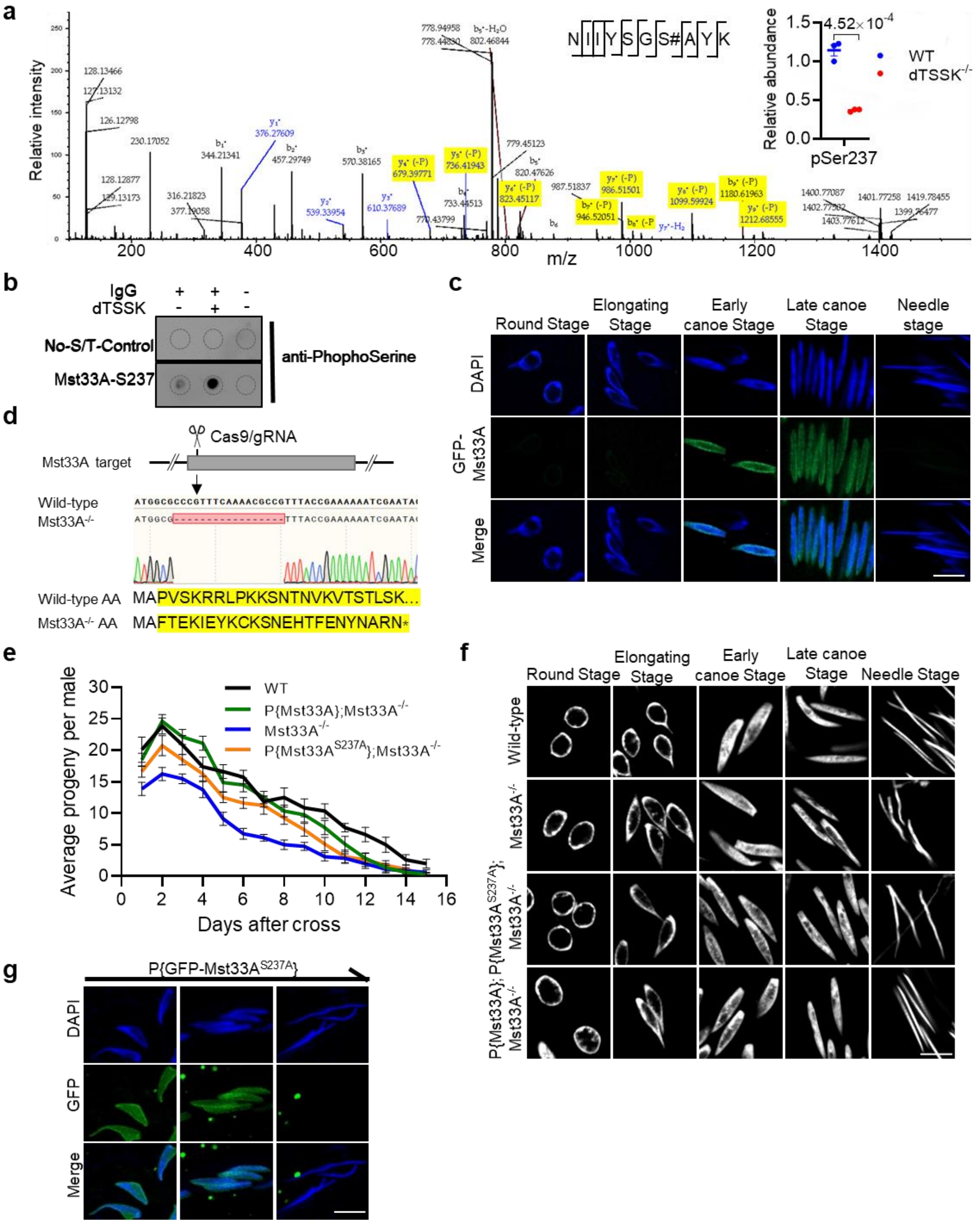

Supplementary Fig. S15. Mst33A is a potential substrate of dTSSK

a Detection of specific y and b fragment ions allowed identification of the peptide sequence NIIYSGSAYK and assignment of the phosphorylation site to Mst33A-Ser237. Plots showing the differences in the relative abundance of the phosphopeptide containing Ser237 from Mst33A between wild-type and dTSSK$^{-/-}$ flies (n = 3 per group). Data are mean ± SEM (paired t-test). **b** Dot blot showing that purified dTSSK-Flag phosphorylates the peptide corresponding to Mst33A-Ser237. A commercial antiphosphoserine antibody was used for dot blot detection. **c** Distribution of Mst33A (GFP-Mst33A) protein in sperm nuclei at different stages of development. DNA stained with DAPI, blue; GFP-Mst33A, green. Scale bar, 5 μm. **d** Mst33A$^{-/-}$ flies generated by CRISPR/Cas9 harbor a 17 bp deletion that induces formation of a premature stop codon. **e** Qualitative fertility assay of wild-type, Mst33A$^{-/-}$, and Mst33A- and Mst33A^{S237A}-rescued Mst33A$^{-/-}$ male flies (n = 8 per group). Data are mean ± SEM. **f** Sperm nuclear morphology at different stages of spermiogenesis in testes of wild-type, Mst33A$^{-/-}$, and Mst33A- and Mst33A^{S237A}-rescued Mst33A$^{-/-}$ flies. DNA stained with DAPI, white. Scale bar, 5 μm. **g** Localization of Mst33A^{S237A} protein at different stages of sperm development. DNA stained with DAPI, blue; GFP-Mst33A^{S237A}, green. Scale bar, 5 μm. Source data are provided as Source Data file.

a

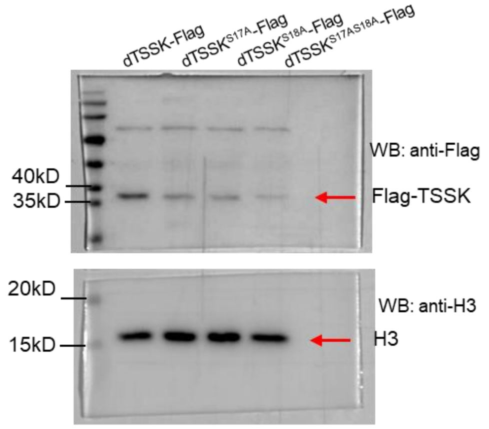

b

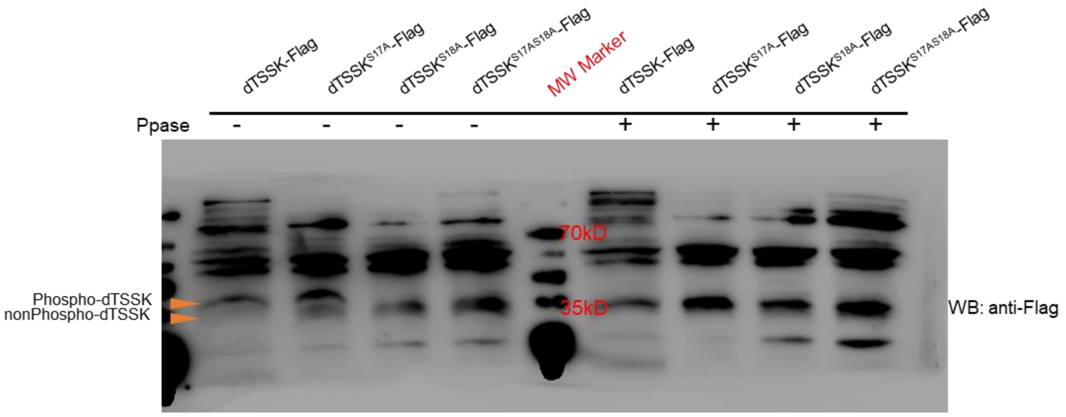

Supplementary Figure 16. Autophosphorylation of dTSSK-Ser18 is essential for spermiogenesis

a Raw image of western blot in Figure 8b. **b** Raw images of western blot in Figure 8c.

Table S1. Potential substrates of dTSSK kinase associated with male-sterile.

Protein name	Phosphorylation site*	Sequence window
Mhc	S1927	[R].AGSVGRGASPAPR.[A]
Up	T27	[R].EETKPPQTPAEGEGDPEFIK.[R]
Sls	S5202	[K].LVQQQSFIEEAQR.[Q]
	S8550	[K].ELSIPTVSPLEAR.[A]
	S2186	[R].SMILDTQHPDALEK.[I]
	T15759	[K].TPVQEVTEEAK.[I]
Ulp1	S1115	[R].QENEENRSPAPQQPK.[R]
didum	S967	[K].TSEISVLK.[M]
	S590; S595	[K].ELTQVLSESNMSLAK.[Q]
	S967; S970	[K].TSEISVLK.[M]
Lasp	S412	[R].SAASVVAYDGNSK.[Q]
	S412; S415	[R].SAASVVAYDGNSK.[Q]
	S504; S505	[R].VVPNAGRSSTTLVYSSEPR.[G]
	S117	[K].HISNVAYHGDLEK.[K]
	S504;S505;T507	[R].VVPNAGRSSTTLVYSSEPR.[G]
	S530	[R].IGSVSDIDPANGIYGSLTAAEQAHQQQK.[H]
Pif1A	S360	[K].QLADTLLSQSPSEVSLQSK.[E]
	S453	[K].KSSYVSVVTSSCR.[S]
	S450	[K].KSSYVSVVTSSCR.[S]
	T790	[R].SNSTLFR.[A]
Fas3	S458	[K].SAAATAAAVIAAGNGSQHNLNGIEAPKPPNTSV.[-]
hts	S1442	[R].VISSTTFVCR.[R]
Ref(2)p	S356; T364	[K].IIDTTEGGDSGIFAPSTTPSAENK.[K]
CIAPIN1	S135	[K].NASAVNVWK.[I]
Pkd2	S189	[K].KTVGSSTPPSPSR.[G]
Osbp	S372	[K].ALPAKESTDSIYGR.[N]
	S369	[K].ALPAKESTDSIYGR.[N]
Cmb	T482; T484	[K].EKEETPTPPPKPEEAAPAAPK.[E]
	S1253; S1256	[K].VPAKPTTSTVSLTPLQQR.[H]
Taf6	S533	[K].FVIVTQNSPQQGQAK.[V]
Dhc16F	S98	[R].ASDANDGSITGPADGNGK.[K]
	S92; S98	[R].ASDANDGSITGPADGNGK.[K]
	S129	[R].LNFSTSK.[S]
Sod	S101	[K].VNITDSK.[I]
	S913; S917	[R].NQLVHSEQNSLK.[I]
kl-3	S29	[R].RPSVSDQER.[E]
Spd-2	S614	[K].NLSPLSSPR.[S]
Fmr1	S577; S578	[R].EMSSVER.[AD]
Exu	S231	[K].EEDAACSASTSGSGSGLGSGSSMVSDSVSISPR.[D]
Su(s)	S202	[R].GGSPPPGGAAPPLSSCGQR.[F]
Eif4G2	S533	[K].QSVSLEKVPEAVYCK.[D]
Klhl10	S621	[R].SALSANNIAGLPNK.[R]
Ran	S135	[K].SIVFHR.[K]
Orb2	S422;S425;S428	[R].LSPHSPHSPIQGGNGGNVGDGTAR.[F]
Gld2	S485; S486	[K].RPSSSSSISPIK.[H]
Grip75	S210	[K].FTPTDAVDGSSFSK.[S]
Dpy-30L2	S4	[-].MPVSPGEGEVNGGGDVAK.[N]
CG17083	S534	[R].LHSISQCR.[L]
Mil	S22	[K].MESDLNFTEK.[E]
Mst77F	S9	[K].QKDSKPEVAVTK.[S]
	S51	[R].AEDEYASSSGFVNFLR.[D]
Ms(3)76Cc	S142; S143	[R].ASSDVNQGHDDPDEHK.[L]
	S53	[R].TEPSCDHWK.[F]
	S755; S756; S759	[R].EVGGRSSNRSQNNTLGR.[S]
	S17	[R].FASAPGSCPR.[I]
	S4	[-].MPISQHDLAR.[F]

*Means significant down-regulated phosphorylation site in dTSSK$^{-/-}$ mutant flies. (> 1.5-fold, P < 0.05). Statistical analyses were performed by two-sided Student’s t-test.
