# Supplementary material for: Broad phosphorylation mediated by testis-specific serine/threonine kinases contributes to spermiogenesis and male fertility
Source: Nat Commun. 2023 May 6;14:2629. doi: 10.1038/s41467-023-38357-0 (PMC10164148; doi:10.1038/s41467-023-38357-0)
Supplement: Supplementary file 3 — Description of Additional Supplementary Files [file 41467_2023_38357_MOESM3_ESM.pdf]

Description of Additional Supplementary Files

File Name: Supplementary Data 1
Description: The significantly down-regulated phosphorylation sites in dTSSK$^{-/-}$ mutant flies.

File Name: Supplementary Data 2
Description: Proteome analysis between wild-type and dTSSK$^{-/-}$ mutants.

File Name: Supplementary Data 3
Description: Oligonucleotides used in this study.

File Name: Supplementary Data 4
Description: Flies used in this study.
