# Supplementary material for: Broad phosphorylation mediated by testis-specific serine/threonine kinases contributes to spermiogenesis and male fertility
Source: Nat Commun. 2023 May 6;14:2629. doi: 10.1038/s41467-023-38357-0 (PMC10164148; doi:10.1038/s41467-023-38357-0)
Supplement: Supplementary file 8 — Reporting Summary [file 41467_2023_38357_MOESM8_ESM.pdf]

Corresponding author(s): Guanjun Gao
Last updated by author(s): Apr 17, 2023

Reporting Summary

Nature Portfolio wishes to improve the reproducibility of the work that we publish. This form provides structure for consistency and transparency in reporting. For further information on Nature Portfolio policies, see our Editorial Policies and the Editorial Policy Checklist.

Statistics

For all statistical analyses, confirm that the following items are present in the figure legend, table legend, main text, or Methods section.

n/a	Confirmed	
☐	☒	The exact sample size (*n*) for each experimental group/condition, given as a discrete number and unit of measurement
☐	☒	A statement on whether measurements were taken from distinct samples or whether the same sample was measured repeatedly
☐	☒	The statistical test(s) used AND whether they are one- or two-sided<br>*Only common tests should be described solely by name; describe more complex techniques in the Methods section.*
☒	☐	A description of all covariates tested
☐	☒	A description of any assumptions or corrections, such as tests of normality and adjustment for multiple comparisons
☐	☒	A full description of the statistical parameters including central tendency (e.g. means) or other basic estimates (e.g. regression coefficient) AND variation (e.g. standard deviation) or associated estimates of uncertainty (e.g. confidence intervals)
☐	☒	For null hypothesis testing, the test statistic (e.g. *F*, *t*, *r*) with confidence intervals, effect sizes, degrees of freedom and *P* value noted<br>*Give P values as exact values whenever suitable.*
☒	☐	For Bayesian analysis, information on the choice of priors and Markov chain Monte Carlo settings
☒	☐	For hierarchical and complex designs, identification of the appropriate level for tests and full reporting of outcomes
☒	☐	Estimates of effect sizes (e.g. Cohen's *d*, Pearson's *r*), indicating how they were calculated

Our web collection on statistics for biologists contains articles on many of the points above.

Software and code

Policy information about availability of computer code

Data collection	Zeiss LSM980 or Leica SP8 confocal system was used to take immunofluorescence images.<br>GE AI680UV instrument was used to take Western Blot images.<br>LC-MS-ThermoScientific was used to collect LC-MS data.
Data analysis	GraphPad Prism8, Image J2.0.0, Mega11, ProteomeDiscoverer™ Software 2.4, MaxQuant with integrated Andromeda search engine (v.1.5.4.1), Gephi v0.9.2, g:Profiler, UniProt, motif-x (v1.2 10.05.06), and the STRING (v11.5)

For manuscripts utilizing custom algorithms or software that are central to the research but not yet described in published literature, software must be made available to editors and reviewers. We strongly encourage code deposition in a community repository (e.g. GitHub). See the Nature Portfolio guidelines for submitting code & software for further information.

Data

Policy information about availability of data

All manuscripts must include a data availability statement. This statement should provide the following information, where applicable:

- Accession codes, unique identifiers, or web links for publicly available datasets
- A description of any restrictions on data availability
- For clinical datasets or third party data, please ensure that the statement adheres to our policy

The mass spectrometry proteomics data have been deposited to the ProteomeXchange Consortium (http://www.proteomexchange.org/) via the PRIDE partner repository with the dataset identifier PXD038767 (https://proteomecentral.proteomexchange.org/cgi/GetDataset?ID=PXD038767) and PXD041466 (https://proteomecentral.proteomexchange.org/cgi/GetDataset?ID=PXD041466). The remaining data are available within the Article, Supplementary Information or Source Data file. Source data are provided with this paper.

Human research participants

Policy information about studies involving human research participants and Sex and Gender in Research.

Reporting on sex and gender	N/A
Population characteristics	N/A
Recruitment	N/A
Ethics oversight	N/A

Note that full information on the approval of the study protocol must also be provided in the manuscript.

Field-specific reporting

Please select the one below that is the best fit for your research. If you are not sure, read the appropriate sections before making your selection.

☒ Life sciences ☐ Behavioural & social sciences ☐ Ecological, evolutionary & environmental sciences

For a reference copy of the document with all sections, see nature.com/documents/nr-reporting-summary-flat.pdf

Life sciences study design

All studies must disclose on these points even when the disclosure is negative.

Sample size	We did not perform statistical methods to predetermine sample size. The sample sizes were determined based on preliminary data or previous reports (refer to PMID: 30741974 and PMID: 35963938) and sample size of each experiment was provided in figure legends. The sample sizes are sufficient to evaluate the effect.
Data exclusions	Data were not excluded.
Replication	All experiments were repeated independently at least twice with similar results.
Randomization	Flies were assigned randomly and in vitro studies were randomly assigned to each group.
Blinding	Investigator was blinded to group allocation and data collection in in vivo experiments. Blinding is not applicable for in vitro experiments in this study, because the same investigator performed flies culture, treatment and data analysis.

Reporting for specific materials, systems and methods

We require information from authors about some types of materials, experimental systems and methods used in many studies. Here, indicate whether each material, system or method listed is relevant to your study. If you are not sure if a list item applies to your research, read the appropriate section before selecting a response.

Materials & experimental systems

n/a	Involved in the study	
☐	☒	Antibodies
☐	☒	Eukaryotic cell lines
☒	☐	Palaeontology and archaeology
☐	☒	Animals and other organisms
☒	☐	Clinical data
☒	☐	Dual use research of concern

Methods

n/a	Involved in the study	
☒	☐	ChIP-seq
☒	☐	Flow cytometry
☒	☐	MRI-based neuroimaging

Antibodies

Antibodies used

Anti-lamin antibody DSHB Cat# LC28.26 Dilution for WB 1:1000
Anti-Histone H3 antibody Abcam Cat# ab1791 Dilution for WB 1:1000
Anti-V5 antibody Invitrogen Cat# MA5-15253 Dilution for WB 1:1000
Anti-Flag antibody Millipore Cat# F7425 Dilution for WB 1:1000
Anti-phosphoserine antibody Merck Cat# 05-1000 Dilution for WB 1:1000
Anti-dTSSK antibody This paper N/A Dilution for WB 1:2000
Anti-Mst77F-pSer9 antibody This paper N/A Dilution for WB 1:5000; Dilution for IF 1:500
Peroxidase AffiniPure Goat Anti-Mouse IgG(H+L) Yeasen Cat# 33201ES60 Dilution for WB 1:5000
Peroxidase-Conjugated Goat Anti-Rabbit IgG (H+L) Yeasen Cat# 33101ES60 Dilution for WB 1:5000
Goat anti-Mouse IgG (H+L) Highly Cross-Adsorbed Secondary Antibody, Alexa Fluor™ 488 Invitrogen Cat# A-11029 Dilution for IF 1:500
Goat anti-Rabbit IgG (H+L) Cross-Adsorbed Secondary Antibody, Alexa Fluor™ 568 Invitrogen Cat# A-11036 Dilution for IF 1:500

Validation

Each commercial antibody is used extensively in our lab and is used based on website verification and our own in house analyses.
Anti-lamin antibody DSHB Cat# LC28.26 is validated by https://dshb.biology.uiowa.edu/LC28-26
Anti-Histone H3 antibody Abcam Cat# ab1791 is validated by https://securedrtest.abcam.com/products/primary-antibodies/histone-h3-antibody-nuclear-marker-and-chip-grade-ab1791.html
Anti-V5 antibody Invitrogen Cat# MA5-15253 is validated by https://www.thermofisher.cn/cn/zh/antibody/product/V5-Tag-Antibody-clone-E10-V4RR-Monoclonal/MA5-15253
Anti-Flag antibody Millipore Cat# F7425 is validated by https://www.sigmaaldrich.cn/CN/zh/product/sigma/f7425
Anti-phosphoserine antibody Merck Cat# 05-1000 is validated by https://www.sigmaaldrich.cn/CN/zh/product/mm/051000
Anti-dTSSK antibody is validated in this study
Anti-Mst77F-pSer9 antibody is validated in this study
Peroxidase AffiniPure Goat Anti-Mouse IgG(H+L) Yeasen Cat# 33201ES60 is validated by https://www.yeasen.com/products/detail/407
Peroxidase-Conjugated Goat Anti-Rabbit IgG (H+L) Yeasen Cat# 33101ES60 is validated by https://www.yeasen.com/products/detail/319
Goat anti-Mouse IgG (H+L) Highly Cross-Adsorbed Secondary Antibody, Alexa Fluor™ 488 Invitrogen Cat# A-11029 is validated by https://www.thermofisher.cn/cn/zh/antibody/product/Goat-anti-Mouse-IgG-H-L-Highly-Cross-Adsorbed-Secondary-Antibody-Polyclonal/A-11029
Goat anti-Rabbit IgG (H+L) Cross-Adsorbed Secondary Antibody, Alexa Fluor™ 568 Invitrogen Cat# A-11036 is validated by https://www.thermofisher.cn/cn/zh/antibody/product/Goat-anti-Rabbit-IgG-H-L-Highly-Cross-Adsorbed-Secondary-Antibody-Polyclonal/A-11036

Eukaryotic cell lines

Policy information about cell lines and Sex and Gender in Research

Cell line source(s)	Drosophila S2 cell line (Thermo Fisher Scientific, R69007) was used in this study.
Authentication	All cell lines were not authenticated.
Mycoplasma contamination	The cell lines were tested negative for mycoplasma contamination
Commonly misidentified lines (See ICLAC register)	No cell line used in the study was found in the databases of commonly misidentified cell lines that are maintained by ICLAC.

Animals and other research organisms

Policy information about studies involving animals; ARRIVE guidelines recommended for reporting animal research, and Sex and Gender in Research

Laboratory animals	Drosophila Melanogaster (fruit flies) were used in these experiments.

Wild animals	Drosophila Melanogaster belong to the order Diptera of the class Insecta. Therefore, this study did not involve wild animals.
Reporting on sex	This study only used fruit flies (insects) and did not involve humans and vertebrate animals sex.
Field-collected samples	The study did not involve samples collected from the field.
Ethics oversight	No ethical approval was required since fruit flies are not typically considered to raise ethical issues related to animal welfare.

Note that full information on the approval of the study protocol must also be provided in the manuscript.
